# Is **β**-lactamase testing acceptably accurate for predicting *Haemophilus influenzae* susceptibility to **β**-lactams? Epidemiological data from Philadelphia, USA, 2017–2023

**DOI:** 10.1128/spectrum.01292-23

**Published:** 2023-09-06

**Authors:** Arianna B. Morton, Chairut Vareechon, Matthew A. Pettengill

**Affiliations:** 1 Department of Pathology and Genomic Medicine, Thomas Jefferson University Hospital, Philadelphia, Pennsylvania, USA; Laboratory Corporation of America Holdings, Burlington, North Carolina, USA

**Keywords:** *Haemophilus influenzae*, BLNAR, β-lactam, AST

## LETTER

*Haemophilus influenzae* is a common cause of infections, including sinusitis, otitis media, respiratory tract infections, and less commonly, bacteremia or central nervous system infections. *H. influenzae* may be resistant to commonly used antibiotics such as ampicillin and amoxicillin by means of a β-lactamase, or additionally, amoxicillin-clavulanic acid and cefuroxime may be resistant via alternate penicillin-binding proteins (PBPs), or both mechanisms may be present. β-Lactamase-negative and ampicillin resistant (BLNAR) *H. influenzae* rates have been poorly documented in the United States, and leading textbooks in the area of clinical microbiology (1) and infectious diseases (2) specifically recommend only performing β-lactamase (nitrocefinase) testing for *H. influenzae* isolates, which cannot detect BLNAR, or infer that for β-lactamase-negative isolates, antimicrobials such as amoxicillin-clavulanic acid and cefuroxime should be active. Limited studies have reported prevalence between 0.0% and 4.1% with regional variation (3 – 6), and one of the recent studies noted that in addition to the 4.1% BLNAR rate, they identified 5.1% of isolates that were β-lactamase-negative and ampicillin-intermediate, which they termed BLNAR-low (6).

**Fig 1:**
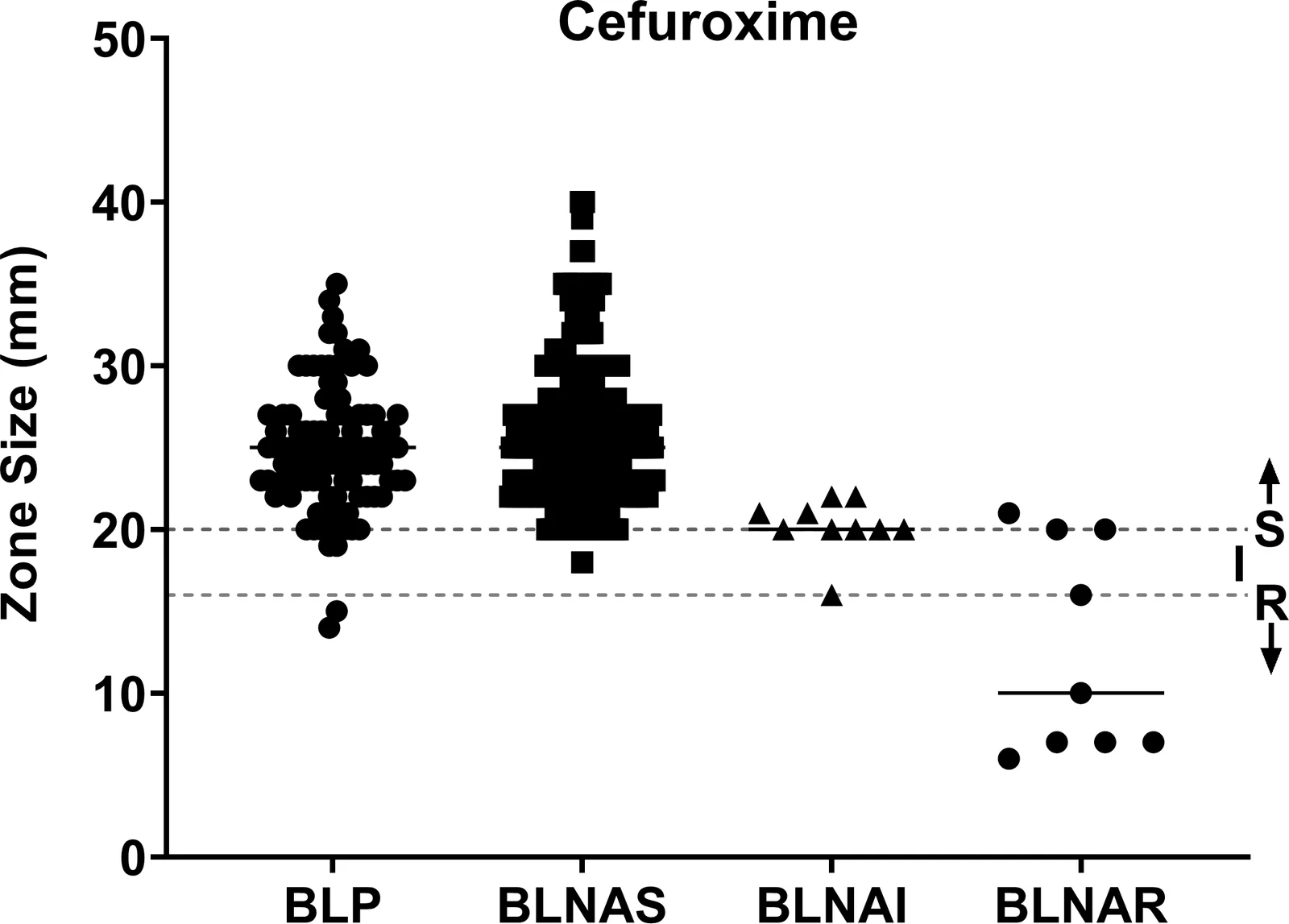
Disk diffusion zone size and CLSI interpretation delineation (M100, 33 edition, ≥20 mm susceptible, 17–19 mm intermediate, ≤16 mm resistant) for cefuroxime for BLP, BLNAS, BLNAI, and BLNAR isolates.

We present here BLNAR rates and antimicrobial resistant patterns among *H. influenzae* isolates at the Thomas Jefferson University Hospital, from April 2017 to June 2023, in part to make local epidemiological data available but also to use our BLNAR rate as a discussion point regarding *H. influenzae* susceptibility testing. We retrospectively extracted data for all *H. influenzae* positive cultures performed in our lab from 2 April 2017 to 15 June 2023, a total of 581 positive cultures. Isolates were excluded if they were a repeat positive culture from the same patient within 12 mo of a previous positive (95 isolates), had incomplete or no testing [37 isolates, missing β-lactamase or ampicillin disk result, or no testing due to patient being deceased prior to antimicrobial susceptibility testing (AST)], or were from certain ocular sources for which we do not test ampicillin (42 isolates). The remaining 407 unique patient isolates of *H. influenzae* had β-lactamase production results by a chromogenic cephalosporin spot test (nitrocefinase) and AST performed by disk diffusion following CLSI M100 methods with the following drugs: ampicillin, ceftriaxone, cefuroxime, levofloxacin, trimethoprim/sulfamethoxazole, and tetracycline, shown separately for each of the following categories: β-lactamase-positive (BLP), β-lactamase-negative ampicillin-susceptible (BLNAS), β-lactamase-negative ampicillin-intermediate (BLNAI, disk zones 19–21 mm), and BLNAR (Table 2). Of the 407 included isolates, 33 were from blood cultures, 331 were from respiratory sources (predominantly sputum), and 43 were from various other sources (<5 from any specific source). *Haemophilus* test media for disk diffusion testing were acquired from Remel (Lenexa, Kansas, USA) and Hardy Diagnostics (Santa Maria, California, USA), antibiotic disks and nitrocefinase β-lactamase tests from Becton Dickinson BBL (Franklin Lakes, New Jersey, USA), and isolates were identified by Bruker MALDI-ToF Mass Spectrometry (Billerica, Massachusetts, USA). In total, nine isolates (2.2%) of BLNAR *H. influenzae* were recovered, which is consistent with other US studies, although it must be emphasized that these numbers vary somewhat regionally. Comparing nitrocefinase β-lactamase results to ampicillin disk diffusion, categorical agreement was high at 95.3%. However, excluding the BLNAI isolates, BLNAR were 9/119-resistant isolates, thus using β-lactamase production to infer ampicillin activity in our study set would have resulted in a very major error (VME) rate of 7.6%. Including BLNAI would mean that 14.7% (19/129) of all isolates non-susceptible to ampicillin in phenotypic testing were negative by β-lactamase testing. Table 1 shows a standard 2 × 2 diagnostic test comparison table for nitrocefinase β-lactamase test results and ampicillin disk diffusion determination of susceptibility or non-susceptibility, indicating that nitrocefinase β-lactamase testing was only 85.3% sensitive for detecting ampicillin non-susceptibility in our study isolates. Considering negative nitrocefinase β-lactamase test results, a prediction of susceptibility to antibiotics such as ampicillin/amoxicillin/amoxicillin-clavulanate/cefuroxime may lead to implied VME rates far in excess of what would be considered acceptable for a commercial test system with the U.S. Food and Drug Administration (FDA) [(7) <1.5% VME] or the recommendations of CLSI guidance document M52 [(8) <3% VME]. Additionally, BLNAR and BLNAI isolates have reduced susceptibility to cefuroxime, and a small number of BLP isolates appear to also have PBP mutations leading to cefuroxime resistance (Fig. 1). Susceptibility to ceftriaxone and levofloxacin was almost uniform in our study set (Table 2). There was also an association for reduced susceptibility to trimethoprim-sulfamethoxazole for BLP isolates (58% susceptible among 110 isolates) compared to β-lactamase-negative isolates (BLNAS, BLNAI, and BLNAR 70% susceptible among 293 isolates; unpaired *t*-test *P* = 0.03, using GraphPad Prism version 9.1.2).

**TABLE 1:** 2 × 2 diagnostic test comparison for ampicillin disk diffusion and nitrocefinase B-lactamase testing among 407 *H*. *influenzae* isolates

β-Lactamase	Ampicillin disk	
	Non-susceptible	Susceptible
+	110	0
−	19	278

**TABLE 2:** AST results for ceftriaxone, cefuroxime, levofloxacin, tetracycline, and trimethoprim-sulfamethoxazole^*a*^

Category	Ceftriaxone	Cefuroxime	Levofloxacin	Tetracycline	Trimethoprim-sulfamethoxazole
BLNAR	88.9% (8 S and 1 NS)	33.3% (3 S and 6R)	100.0% (8 S)	77.8% (7 S and 2 R)	77.8% (7 S and 2 R)
BLNAI	100.0% (10 S)	90.0% (9 S and 1R)	100.0% (8 S)	70.0% (7 S and 3 I)	60.0% (6 S and 4 R)
BLNAS	100.0% (278 S)	99.6% (273 S and 1 I)	100.0% (260 S)	85.6% (238 S, 30 I, and 10 R)	69.7% (191 S, 2 I, and 81 R)
BLP	100% (110 S)	98.1% (103 S, 2 I, and 2 R)	99.0% (100 S and 1 NS)	81.8% (90 S, 18 I, and 2 R)	58.2% (64 S and 46 R)

^*a*^ Shown as percent susceptible and in brackets category isolate counts: S, susceptible; NS, non-susceptible; I, intermediate; R, resistant.

We suggest evaluating local rates of BLNAR/BLNAI *H. influenzae* to determine if performing AST in addition to nitrocefinase β-lactamase testing is justified.
